# Application and challenges of using a Constructivist Grounded Theory methodology to address an undertheorized clinical challenge: A discussion paper

**DOI:** 10.1016/j.ijnsa.2024.100199

**Published:** 2024-04-10

**Authors:** Paul Bobbink, Philip Larkin, Sebastian Probst

**Affiliations:** a HES-SO, University of Applied Sciences and Arts, Western Switzerland, Geneva School of Health Sciences. Geneva, Switzerland; b University Institute of Higher Education and Research in Healthcare, Faculty of Biology and Medicine, University of Lausanne, Lausanne, Switzerland; c Department of Palliative and Supportive Care and Institute of Higher Education and Research in Healthcare-IUFRS, Lausanne University Hospital, University of Lausanne, Switzerland; d Care Directorate, University Hospital Geneva, Switzerland; e College of Medicine Nursing and Health Sciences. University of Galway, Ireland; f Faculty of Medicine Nursing and Health Sciences, Monash University, Melbourne, Australia

**Keywords:** Grounded theory, Nursing methodology research, Patient education as topic, varicose ulcer

## Abstract

**Background:**

The benefits of nurse-led therapeutic patient education regarding wound healing and the prevention of recurrences for individuals living with a venous leg ulcer remain unclear. Obtaining the individuals perspective could offer an in depth understanding of why and how they engage or not, in self-management strategies following nurse-led patient education. Despite strong evidence indicating the need for further investigation into the benefits of therapeutic patient education in this population there is a lack of research into how individuals cope with chronic venous insufficiency or resulting ulceration. With this discussion paper we therefore explore the challenges associated with employing a Constructivist Grounded Theory methodology to gain a deeper insight into the experiences of patients with venous leg ulcers receiving individualized nurse-led patient education programs focused on the self-management of their condition.

**Objective:**

To identify and analyse the specific methodological and practical challenges encountered when applying a Constructivist Grounded Theory methodology to gain a better understanding of how patients with venous leg ulcer experience an individualised nurse-led patient education programme concerning the self-management of their condition.

**Design:**

discussion paper

**Results and Discussion:**

The constructivist approach to Grounded Theory methodology allows for the investigation of understudied phenomena such as nurse-led patient education for individuals living with venous leg ulcers. This methodology values the co-construction of a theory taking into consideration the inherent value of participants’ and researchers’ experiences. However, the specificities of constructivist epistemology challenge certain methodological aspects of Grounded Theory methods, such as how and when to use existing literature, conduct interviews to generate data and engage in the coding and theoretical sampling process for conceptualizing and proposing a theory.

**Conclusions:**

The constructivist paradigm of grounded theory methodolgy resonates with the art and science of nursing through its collaborative ‘real-world’ reflective approach, offering a unique way to explore understudied complex clinical nursing practice.

**Registration:**

This methodological paper is derived from a PhD study embedded in a clinical trial (NCT04019340) were the recruitment started on February 2020, approved by ethical committee of Geneva (CCER: 2019-01964)

**Tweetable abstract:**

Constructivist Grounded Theory Methodologies could support an in depth understanding of the impact of nursing interventions

## Abstract (author highlights)

What is already known

• Effectiveness of nurse-led patient education for individuals living with a venous leg is unknown
• Grounded Theory Methodology is well-known for developing theories grounded in the data.
• Constructivist Grounded Theory methodology challenges certain aspects of the Grounded Theory Method.

What this paper adds

• This paper provides a clear overview of how to use existing literature and interviews to generate data and develop theory using a Constructivist Grounded Theory methodology
• This paper discusses how a theory could merge from a co-construction between the researcher and the participants.

## Introduction

In recent decades, the increasing number of people living with chronic health conditions has become one of the critical challenges for healthcare systems worldwide (World Health Organization, 2023). To face these challenges, a variety of disease-specific nurse-led therapeutic patient education programs have been developed. These programs aim to enable those living with chronic disease to self-manage their illnesses, thereby improving their quality of life while reducing healthcare costs (World Health Organization, 1998). The concept of therapeutic patient education varies across settings, contexts, or disciplines and is often used interchangeably with other concepts such as health education or patient information (Pueyo-Garrigues et al., 2019).

Whereas clinical trials highlight the efficacy of therapeutic patient education to promote health and financial benefits in multiple chronic conditions including diabetes, respiratory or circulatory system disease (Correia et al., 2022; Simonsmeier et al., 2022), there is unclear evidence of their efficacy when used with individuals living with venous leg ulcers (Shanley et al., 2020; Weller et al., 2016).

Venous leg ulcers are open skin lesions on the leg or foot that result in an area affected by venous hypertension (O'Donnell et al., 2014). This chronic condition affects approximately 0.32 % of the global population and has a broad impact on health-related quality of life (Probst et al., 2023). This can also affect the therapeutic patient education process and individuals’ ability to engage in self-management. The aetiology of venous leg ulcers appears to be misunderstood by those affected (Phillips et al., 2018) and recent evidence suggests people living with a venous leg ulcer are at risk of, or have to cope with multiple other chronic conditions (Gethin et al., 2021; Kelly and Gethin, 2019). Impairments related to venous leg ulcers are often physical or psychological such as pain, decreased mobility, or social restrictions (Phillips et al., 2018). However, a recent literature review highlights that individuals living with a venous leg ulcer also feel guilty when imposing the burden of their condition on family or friends (Klein et al., 2021).

To prevent recurrences and manage and treat venous leg ulcers, various guidelines recommend self-care treatments, such as wearing compression therapy (Tan et al., 2019), practicing specific exercises, and following a particular diet (Probst et al., 2019).

The literature emphasizes the close relationship between nurses and individuals living with venous leg ulcers (Klein et al., 2021) but only a few studies have explored the effectiveness of individualized nurse-led educational interventions for promoting self-management among those with venous leg ulcers (Bobbink et al., 2020). A recent study highlighted that recurrence was not necessarily linked to a decrease in self-efficacy scores or quality of life. Hence, nonadherence to therapy could not be the primary hypothesis for venous leg ulcer recurrence (Probst et al., 2021). This under-researched area warrants further exploration to gain a deeper qualitative understanding of the self-management journey of individuals living with venous leg ulcers. Evidence concerning the utilization of Grounded Theory methodology to comprehend the processes of engagement in self-management for individuals with chronic conditions is limited. In the management of patients with diabetic foot ulcers, a Constructivist Grounded Theory methodology can conceptualize how patients acquire specific practices and become engaged in day-to-day management (Costa et al., 2021). Even though evidence indicates that the benefits of therapeutic patient education in this population warrant further investigation the manner in which individuals cope with chronic venous insufficiency or a resulting ulceration remains under-researched (Shanley et al., 2020; Weller et al., 2016).

Therefore, our aim is to discuss the challenges of utilizing a Constructivist Grounded Theory methodology to gain a better understanding of how patients with venous leg ulcer experience an individualised nurse-led patient education programme concerning the self-management of their condition.

### The evolution of constructivist grounded theory methodology

Grounded Theory methodologies provide unique opportunities for an in-depth analysis of the underlying processes of a phenomenon and have the potential to develop theories related to social interactions and complex relationships among individuals (Tarozzi, 2020). These methodologies are typically classified as a qualitative approach due of the nature of the data collected, which includes a range of observational methods, individual interviews, or focus groups (Charmaz, 2014; Glaser and Strauss, 1967). However, in certain approaches to Grounded Theory, quantitative approaches can also be incorporated (Glaser and Strauss, 1967). As a result, Grounded Theory methodologies transcend the discourse on qualitative versus quantitative approaches and serves as a unique inductive, systematic and comparative method for generating theories grounded in the data (Bryant, 2017; Charmaz, 2014; Glaser and Strauss, 1967). The Grounded Theory method is situated within methodological pluralisms (Gibson and Hartman, 2014) evolving into three main approaches; the work of Glaser, Corbin and later, Charmaz (Bryant, 2017). The epistemological and ontological differences principally address the proposition that theory is either discovered or co-constructed (Bryant, 2017), leading to opposing views on how the steps in the Grounded Theory method are understood (Bøttcher Berthelsen, 2018; Chun Tie, Birks and Francis, 2019; Tarozzi, 2020). This impact affects both the quality criteria applied to research projects (Bøttcher Berthelsen, 2018) and the outcomes of the resulting grounded theory (Singh and Estefan, 2018).

Contemporary authors in the field recognize the variety of epistemological and ontological standpoints regarding the Grounded Theory methodology. However, they emphasize its shared methodological strengths, including the constant comparative method, simultaneous data collection and analysis, theoretical sampling, and the use of memos or diagrams to promote conceptualisation (Tarozzi, 2020).

The constructivist Grounded theory methodology proposed by Charmaz (2014), guided by an interactionist and pragmatist philosophy, embodies these methodological strengths and values creativity in capturing the voices of patients to better understand their experiences. By placing both the researcher and the subjects within the field of inquiry (Charmaz, 2011) this approach repositions reality as experienced by people (Bryant, 2019) and relies on contextual interpretation including factors such as time and space (Bryant, 2019).

### The use of a constructivist grounded theory methodology to understand patients’ self-management journeys

The Constructivist approach to Grounded Theory methodology, as defined by Charmaz (2014) was selected to develop a comprehensive and contextualised explanation of how patients with venous leg ulcer experience an individualised nurse-led patient education programme concerning self-management. This section will provide a critical discussion of the methodological issues raised by its use.

### Using existing literature

The use of existing literature is a crucial aspect in Grounded Theory methodology (Dunne, 2011; Tarozzi, 2020; Thornberg and Dunne, 2019). Most research projects necessitate a literature review to delineate the existing knowledge within a particular topic. This background literature review is essential for assessing the gap and the intended contribution of the research to the body of knowledge, especially when presenting to institutional review boards or ethics committees. Furthermore, it underscores any existing knowledge deficiencies, missing theories, and highlights the project's relevance. Identifying a gap without conducting a literature review appears incongruent. A literature review not only helps researchers become well-versed in the subject but also fosters the development of Grounded Theories in areas not covered by existing theories.

According to the constructivist worldview, in which theory emerges from co-construction (Tarozzi, 2020), both participants and the researcher influence data based on their backgrounds and life experiences. Due to their previous academic training, researchers can find it challenging to avoid preconceived theories or empirical studies within the domain of interest (Charmaz, 2011). Consequently, a Constructivist Grounded Theory methodology takes a nuanced approach to the use of existing literature during the research process. Depending on the research objectives and its progression, the scope, focus or depth of the literature reviews will vary throughout the process. Illustrations below will further clarify this approach. To support the research project and promote his expertise during the early stages of the study, the first author (PB) recognized a scoping review to map existing evidence on nurse-led therapeutic patient education for individuals living with a venous leg ulcer (Bobbink et al., 2020) was necessary. Scoping reviews are well-suited to examine how research has been previously conducted in the field and identify knowledge gaps (Munn et al., 2022). They offer an appropriate starting point of developing a Constructivist Grounded Theory because they are open-ended, revealing areas where knowledge remains unclear. Charmaz (2014) argued that the literature review should not hinder creativity or constrain the emergence of the theory. A well-structured critique of the literature at the beginning of Constructivist Grounded Theory inquiry can empower the researcher (Thornberg and Dunne, 2019), providing the confidence needed to enter the field and commence data collection.

Throughout both the data collection and analysis phases of the Constructivist Grounded Theory method, the researcher needs to be aware of existing literature to better comprehend and situate participants meanings. During this phase of the study, the need to consult existing literature is guided by data and initial analysis (Bryant, 2019). In doing so, data from other sources can be engaged to understand emerging concepts and refine questions during in-depth interviews. Using venous leg ulcer patient education literature as an example, Protz et al. (2019) discussed the utilization of a brochure to enhance the knowledge of individuals living with venous leg ulcers. Their results suggested that brochures should be used by healthcare providers to address participants’ inquiries. In our study, the intervention involved both face-to-face meetings and an educational brochure. Therefore, Protz et al.s (2019) results assisted the researcher, in the current study, in better guiding and comprehending the in-depth interviews. For instance, they helped focus questions during interviews to identify the specific and contextual contributions of a brochure versus face-to-face meetings with nurses during the participants’ learning process. In the interviews, participants’ concerns underscored the significance and potential variability in how, when, and why a brochure or an individualised interaction was necessary. Integrating both the prior analysis of interviews and results from existing literature critically, stimulates questioning during interviews, enabling comparison, confrontation, and the emergence of new data from participants’ concerns.

Considering the significance of context in constructivism, it is advisable to approach current literature with care, as the context of each investigation can differ. When conducting interviews with individuals regarding their engagement in venous leg ulcer self-management, they might emphasize various barriers or facilitators. A recent qualitative systematic review outlined barriers and facilitators to physical activity for individuals living with a venous leg ulcer (Qiu et al., 2022). Their results showcase similarities in participants’ voices which for the current study, were crucial for gaining a better understanding of the process of engaging in self-management strategies. Their suggested outcomes resonated with the generated data and subsequent emerging categories. However, regarding the constructivism worldview and the importance of the context, the literature was not included and coded into the analysis as interview transcripts. Rather it enhanced probing during interviewing and critically supported confidence in the research project by enabling similarities, divergent views and unexpected knowledge to emerge.

Taking into consideration other sources of data and information supports the enhancement of data analysis at a theoretical level and the initial development of the theory. Integrating additional data helps in theoretical reflection, but this introduces a critical discussion point regarding professional concepts versus participants’ concerns (Gibson and Hartman, 2014).

The reflective utilization of existing research involves a choice between ignoring the literature prior to the analytical core of the category emerged (Glaser and Strauss, 1967) and critically engaging with other findings from the start point (Charmaz, 2014). This situates the Constructivist Grounded Theory methodology between an inductive and abductive approach, which is essential in theory construction. In the final step, referring to existing literature enables the researcher to confront and discuss similarities and differences between the Constructivist Grounded Theory and pre-existing theories. This step, called “*returning to the library*” (Charmaz, 2014), provides the researcher with the chance to situate the theory within various disciplines and advocate for a novel contribution to the chosen disciplinary field. In conclusion, the Constructivist Grounded Theory methodology advocates for searching the literature at different timepoints. The published literature should be utilized in a reflective and critical way throughout the different steps, encompassing background description, data collection, conceptual development, and dissemination, to underpin the entire process. Consequently, the researcher needs to identify when, how and why literature becomes necessary. Nevertheless, when engaging in the Constructivist Grounded Theory method, numerous methodological challenges emerge at different stages. Hence, methodological literature, encompassing epistemological or ontological debates about Grounded Theory methodologies and methods, can aid in enhancing reflexivity to identify strengths and limitations of the method and support the analytical phases of the Constructivist Grounded Theory.

### Interviewing to produce data

Through Interviews, researchers can gain access to social worlds and realities while considering the specific context (Miller and Glassner, 2021). Interview methods are extensively employed in qualitative research and establish an interaction between participants and researchers, placing them both within the field of inquiry. Interviews can be approached as a form of symbolic interaction, enabling an understanding of the meaning's individuals attribute to their experiences (Charmaz, 2014), and providing the opportunity to theorise about the social world (Miller and Glassner, 2021). Within a constructivist worldview, interviews represent an active construction (Tavory, 2020), offering a unique opportunity to capture diverse and rich experiences that can serve as a foundation to co-create reality.

Interviewing with open-ended questions allows for flexibility in data collection and involves the researcher as an integral part of the research project. Charmaz (2014) argued that intensive interviewing aligns best with the Constructivist Grounded Theory method as it permits openness and an in-depth exploration of the subject area. Engaging in a conversational approach during data collection fosters the emergence of diverse realities (Charmaz, 2014). By utilizing this method, the researcher can maintain an open mindset towards participants' meanings, thus comprehending and making sense of their experiences (Miller and Glassner, 2021). A constructivist approach assumes that the researcher influences the process due to their prior understanding and/or engagement in the topic or field. This critical aspect of the Constructivist Grounded Theory methodology approach should not be interpreted as social desirability bias. For instance, in the context of this study, participants frequently discuss the dressings they use for their wounds and anticipate the first author to remember the product's name. Providing them with an answer did not interfere with the research process but it allowed the researcher to become a ‘member of the group’ (Miller and Glassner, 2021) possessing knowledge in the field. As a result, this facilitate gaining confidence from the participants, deepening interactions, strengthening the data, and supporting the view of knowledge as socially constructed (Tarozzi, 2020).

Interviewing through a conversational approach is beneficial for both the researcher in collecting rich data and for the participants, as it could provide them with a learning opportunity. For instance, in this study, one participant said: ‘*it's something that I realise from talking to you that it's something I'm going to have to see with*‘ […] *’by talking to you and the doctor each time, it allows me to put things back in order and to realise that what I do now is essential’* (freely translated from French by PB). According to Miller and Glassner (2021), interviews grant access to meanings and encourage participants’ reflexivity.

In Constructivist Grounded Theory methodology, interviews are conducted not only to describe the worldview but also to facilitate the development of a theory (Charmaz, 2014). As a result, the approach to conducting interviews evolves during the construction of the Constructivist Grounded Theory. For example, when critically evaluating how interviews were carried out in the research project, the interview questions became progressively more precise. This refinement utilized previously analysed data to gather additional perspectives from other participants to develop categories. In the final stage of data generation, to ensure the accuracy of the data, diagrams illustrating the emerging theory are presented and discussed with the participants during interviews. This collaborative approach results in a co-construction of the research outcomes. The use of intensive interviewing allows for the acquisition of a diverse range of data and fine-tunes the data collection process, focussing on the most relevant aspects and advancing the theory's development (Charmaz, 2014; Wimpenny and Gass, 2000). According to Charmaz (2011), the process of revising the interview guide to include focused questions about categories and their properties constitutes a part of theoretical sampling.

Hence, a naturalist point of view emphasizes the significance of genuinely considering subjective experiences (Miller and Glassner, 2021). In the Constructivist Grounded Theory methodology, Charmaz (2014) argued for the necessity of maintaining objectivity towards the generated data while representing the consistently subjective meanings of individuals, ensuring an adequate level of depth and detail. Therefore, it seems advisable to record and transcribe verbatim the interviews.

### Engaging in coding and conceptualisation

Coding holds a central place in Constructivist Grounded Theory methodology and accompanies the entire process of theory construction (Bryant, 2017; Charmaz, 2014). The development of the theory hinges on how researchers examine and analyze the data (Bryant, 2019). Within the Constructivist Grounded Theory method, coding follows a non-linear process (Bryant and Charmaz, 2019) serving as a fundamental component in constructing the Constructivist Grounded Theory. Coding entails more than labelling; thus, participating in the coding process poses challenges for researchers, as it may reveal uncertainties, doubts, or a lack of self-confidence (Bryant, 2019; Tarozzi, 2020). According to Charmaz (2014), the Constructivist Grounded Theory methodology encompasses at least two phases of emergent coding. The initial coding phase involves either word-by-word or line-by-line coding of transcribed interviews, enabling a more profound comprehension (Charmaz, 2014). These preliminary steps permit the researcher to familiarize themselves with the data and concentrate on data fragments and remain open to various potential theoretical directions (Charmaz, 2014). Line-by-line coding might be somewhat arbitrary as it does not include complete sentences (Charmaz, 2014). Consequently, Tarozzi (2020) suggests extracting the smallest text sections that pertain to their meaning. Nevertheless, this initial stage aims to deconstruct / fracture the data, allowing details to emerge (Bryant and Charmaz, 2019) to create the opportunity to identify both similarities and differences, pose analytical inquiries (Charmaz and Thornberg, 2021), and thereby establish to attain a more profound level of meaning (Chun Tie et al., 2019). For a novice grounded theorist, this stage is challenging as the codes are created from the interaction between the researcher and the transcripts aiming to condense meanings and actions (Bryant, 2019; Charmaz, 2011; Gibson and Hartman, 2014). In the context of employing a constructivist worldview, the researcher selects pertinent segments and construct codes (Charmaz, 2014; Rieger, 2019). This active process of assigning labels to data has the potential to generate tensions between data and the researcher's perceptions, even if the researcher maintains the belief that the codes accurately mirror the transcribed events of the interviews.

According to Charmaz (2014), neutrality during coding is not possible because language confers form and meanings to observed realities. During these initial steps, it is recommended to seek actions and process to coding using gerunds (-ing) whenever possible to preserves movement, facilitate abstraction, and illustrate a process (Bryant and Charmaz, 2019; Bryant, 2019; Charmaz, 2014; Chun Tie et al., 2019). As a distinctive feature in Grounded Theory, coding should facilitate the transition to abstraction, including categories and concepts (Belgrave and Seide, 2019; Bryant, 2017).

To enhance the analysis and allow the codes to emerge from the transcribed interviews the researcher should deal from an early stage with their preconceptions and knowledge of the field. During data analysis, the first author followed Charmaz (2014) advice of using line-by-line coding, identifying actions in the data and providing codes that are close to the data.

This initial coding step could yield a very large number of codes, potentially overwhelming the researcher. In this initial phase, Charmaz (2014) and Bryant (2019) recommend to move quickly through the data. To prevent struggling during this step, and drawing upon the experience of the first author, asking support from a second coder can facilitate progress in coding and enhance conceptualisation. However, this contribution should be examined closely, as the objective is not to validate initial codes, but to contemplate the resemblances and disparities and how they might contribute to novel insights (Birks et al., 2019; Charmaz, 2014; Flick, 2019; Tarozzi, 2020). When required during the coding process, in vivo codes, which involve employing participants’ exact words as codes, remain descriptive and faithfully preserve participants’ meanings and perspectives (Charmaz, 2014). Nevertheless, when the data employed are generated from interviews, commencing initial coding early in the research process is essential. This enables the application of the constant comparison process inherent in Grounded Theory methods and provides an opportunity to uncover new areas of investigation (Tarozzi, 2020).

Transition from initial coding to focus coding is not clearly defined in Constructivist Grounded Theory methodology, possibly due to its nonlinear process (Charmaz, 2014; Tarozzi, 2020). The goal of focus coding is to enhance the level of abstraction by reusing previous codes to sort and analyse a larger volume of data. This step allows for conceptualization without sacrificing detail (Charmaz, 2014) and is guided by the significance of the codes, enabling researchers to make informed decisions. These decisions can give rise to tensions between participants and professionals’ meanings (Charmaz, 2014) or previous readings thereby forcing emergence of inadequate categories. To prioritize participants’ concerns over personal or disciplinary concepts, several authors (Bryant, 2019; Charmaz, 2014; Gibson and Hartman, 2014) support the need to explore how one's past influences the interpretation of the data. Gibson and Hartmann (2014), state that preconceptions could pose challenges as they can compel the construction of categories or results in “selective blindness”. For illustration, in the early stages of this study, the analysis of collected data relates to the primary content of the intervention. Considering my preconceptions and extracting relevant meanings from the interview transcription, the construction of categories was “revisited”. To better focus on participants meanings, Charmaz (2014) recommends analysing with the following question: can the data adequately be interpreted on their own? Subsequently, taking Bryant's (2017) suggestion in consideration, the first authors used diagrams to connect ideas that could relate to the same categories.

Reflexivity poses a challenge when employing a qualitative approach. Corlett and Mavin (2018) emphasize the significance of recognizing the researcher as a research tool or as a coder. Grounded Theory methodologies offer a distinctive opportunity to enhance reflexivity through the use of memos (Rieger, 2019). Memos, referred to as a “storehouse of ideas’” (Chun Tie et al., 2019) should be employed at the beginning of the Constructivist Grounded Theory methodology (Charmaz, 2011), and their utilization should expand in conjunction with coding, category sorting, and interlinking (Bryant, 2017; Charmaz, 2014; Coşkun, 2020). These memos, central to Constructivist Grounded Theory construction, play a pivotal role in maintaining reflexivity throughout the research project. They serve to clarify for example what happened during interviews, articulate new concepts or reflections on existing literature, and ensure traceability in theory construction when utilizing constant comparison methods. Constant comparison is a systematic strategy combining the advantages of simultaneous data generation and analysis, enabling a concentration on significant issues (Charmaz and Thornberg, 2021). Though the comparison of data, researchers emphasize consistencies, comprehend differences, and refine interviews, consistently comparing specific data points with others to establish categories (Bryant, 2017; Charmaz, 2014; Chun Tie et al., 2019). Constant comparison is directly connected to coding, facilitating reflexivity and the application of theoretical sampling. Theoretical sampling presents challenges within Grounded Theory methods *per se*, as it enables the inclusion of additional participants, adjustment of recruitment timelines, or refinement of interview questions. For instance, in the context of this research project, the necessity for new data emerged. This led to the consideration of incorporating quantitative data from the project, such as wound size reduction, in the discussions concerning participants’ interpretation of wound healing. Engaging in focus coding contributes to enhancing confidence in the ongoing analysis (Charmaz 2014). Through the comparison of data and the recognition of relationships between categories, focus is directed towards theoretical development (Bryant and Charmaz, 2019; Charmaz, 2014). This dynamic process enables researchers to interact with the data, ensure congruency between categories and the data, and develop the Constructivist Grounded Theory.

Grounded Theory coding process vary across the main approaches, including for example axial coding in Strauss and Corbin's approach or theoretical coding in Glaser's version of Classical Grounded Theory (Belgrave and Seide, 2019). Charmaz (2014) supports the perspective that initial coding and focus coding will often prove adequate for numerous projects. This is because the connections between categories should be articulated directly by the participants descriptions (Gibson and Hartman, 2014). In the Constructivist Grounded Theory method, this variety of coding strategies including among others theoretical coding or axial coding, can be employed if they contribute to theory generation, but they are neither advocated nor discouraged illustrating openness in the Constructivist Grounded Theory Method (Belgrave and Seide, 2019).

### Creating a meaningful constructivist grounded theory to comprehend contextualized nursing practice

The objective of the Constructivist Grounded Theory methodology is to generate a theory that unveils the underlying processes of a phenomenon. This theory is often characterized as a processual, encompassing temporal sequences (Tarozzi, 2020). However, the constructivist Grounded Theory methodology has occasionally met strong criticism for deviating from the intent of the “original” method, which seeks to develop a theory grounded solely in the data and avoids incorporating speculative constructivist elements (Simmons, 2022).

Contrary to the notion that “constructionism is used to legitimate forcing” (Glaser, 2002, cited in Simmons, 2022 p.30), Constructivist Grounded Theory situates itself in the paradox of simultaneously being both open and closed, and is intended to culminate in a theory that interconnects the interpretations and representation of experiences from both participants and researchers (Gibson and Hartman, 2014). The distinctive attributes of Constructivist Grounded Theory methodology are rooted in the notion that knowledge and truth emerge from a co-construction involving both the researcher and participants (Bryant, 2019; Tarozzi, 2020).

A Constructivist Grounded Theory, influenced by pragmatism, should encompass multiples realities, exhibit problem-solving attitudes, and embrace the concept of truth as provisional (Charmaz, 2014). Integrating structured disciplinary concepts and participant concerns into a Constructivist Grounded Theory framework enables the evolution of nursing sciences and practice toward a person-centered approach that considers the desires, preferences and needs of individuals and their families. Consequently, a Grounded Theory holds the potential to offer valuable guidance for nurses within their discipline, based on real-world experiences (Nathaniel and Andrews, 2007). For instance, it can help in understanding the underlying processes behind the implementation of self-management strategies within a specific population and context.

Quality criteria are crucial in research and are contingent upon the epistemological perspectives that shape the study design. As a result, a methodology focused on theory generation cannot be assessed using the identical criteria as research centered around hypotheses testing (Tarozzi, 2020). At this time, the Equator network (2023) offers guidance to enhance the quality of qualitative research through the use of the COnsolidated criteria for REporting Qualitative research (COREQ) checklist (Tong et al., 2007). However, these guidelines might not effectively capture inconsistencies, lack of rigour, or inadequate understanding of the Grounded Theory methods. Due to the paradigmatic distinctions between various Grounded Theory approaches and in order to mitigate unproductive criticism, each approach requires distinct quality criteria (Rieger, 2019). To address this issue, Bøttcher Berthelsen et al. (2018) have developed a guideline for reporting Grounded Theory research that considers diverse epistemological standpoints.

Charmaz’ (2014) defined four criteria for Constructivist Grounded Theory. Credibility, the first criterion, involves encompassing adequate scope of data and depth of analysis. This pertains to the coding process, achieved through comparison between data, coding and theoretical sampling, all of which contribute to obtaining comprehensive insights into participants’ meanings. The second criterion, resonance pertains the Constructivist Grounded Theory's ability to attain a satisfactory theoretical depth. This encompasses the theory's relevance to the individuals involved and its potential for extension to a wider population. In the context of nursing science, development of a theory will provide essential best practice support in delivering nurse-led patient education for individuals living with a venous leg ulcer. Lastly, originality encompasses the new conceptual insights offered by the proposed theory, along with its practical utility for individuals to apply the developed theory (Charmaz, 2014). All four criteria align well with higher academic studies such as a PhD in nursing sciences project, which aims to contribute rigorously to the body of nursing knowledge, with the ultimate aim of improving nursing practice to enhance patient outcomes.

## Conclusions

Since its discovery in 1967, the Grounded Theory method has continued to evolve, remaining a dynamic and contributory method within health research. Constructivist Grounded Theory, in particular, represents an analytic, adaptable and flexible methodology that facilitates the construction of explanatory theories. By theorizing “from the ground” - drawing insights directly from clinical or bedside settings - and accepting the co-construction of the theories, researchers can effectively consider the social context, and focus on processes as they are lived by the individuals involved. This aligns the constructivist paradigm of Grounded Theory method with the essence of nursing, combining both art and science. The collaborative and engaging nature of this approach emphasizes the crucial voice of the individuals undergoing life-changing experiences, and it offers innovative and distinct avenues to explore complex practices that may have been understudied within a specific context.

### Ethics, registration and funding

This methodological paper is derived from a PhD study embedded in a clinical trial (NCT04019340) approved by ethical committee of the canton of Geneva (CCER: 2019-01964). Verbatims used are translated from French to English by the first author. This project is funded by the Swiss 10.13039/100000001National Science Foundation (10531C_185332) and supported by the HES-SO University of Applied Sciences and Arts, Geneva, Switzerland.

## CRediT authorship contribution statement

**Paul Bobbink:** Writing – original draft, Conceptualization, Methodology. **Philip Larkin:** Writing – review & editing, Supervision, Methodology. **Sebastian Probst:** Writing – review & editing, Supervision, Project administration, Methodology, Funding acquisition.

## Declaration of competing interest

The authors declare that they have no known competing financial interests or personal relationships that could have appeared to influence the work reported in this paper.

## Appendix Supplementary materials

Image, application 1

Image, application 2

Image, application 3
