# Supplementary material for: Application and challenges of using a Constructivist Grounded Theory methodology to address an undertheorized clinical challenge: A discussion paper
Source: Int J Nurs Stud Adv. 2024 Apr 10;6:100199. doi: 10.1016/j.ijnsa.2024.100199 (PMC11080569; doi:10.1016/j.ijnsa.2024.100199)
Supplement: Supplementary file 2 [file mmc2.doc]

**IJNS author checklist V 3.0 (August 2021)**

| **IJNS AUTHOR CHECKLIST:** You will need to submit a completed version of this checklist plus the checklist from any relevant reporting guideline along with your paper. It is intended to help you to make sure your manuscript meets some basic requirements for the journal. It should be read in conjunction with the guide for authors, and is not a replacement for it. Additionally, to help ensure your manuscript is compliant, a manuscript template is available (linked from the guide for authors). | | Insert a tick, page number(s) or give detail |
| --- | --- | --- |
| **Word count** | The paper is 7000 words or fewer | 6252 |
| **Abbreviations** | No abbreviations (including acronyms or “initialisms”) are used anywhere in the paper (other than SI units, common statistical terms and other limited exceptions identified in the guide for authors). | Ok, Except WHO according to the journal preferences |
| **Reporting guideline** | The paper has been prepared using a recognized reporting guideline appropriate to the method / type of paper. Please consult <https://www.equator-network.org/> to help select an appropriate guide [research and reviews only]. **Please identify the reporting guideline used in the box to the right.** | Not applicable for discussion papers |
|  | A checklist linked to the reporting guideline should be completed and uploaded as part of your submission. *If there is no applicable guideline, upload a blank file with the words ‘not applicable’ when requested at submission.* | Not applicable for discussion papers |
|  | Do not refer to *reporting* guidelines as a source for your *methods.* (i.e. the manuscript should not contain a statement such as “This trial was conducted according tothe CONSORT guideline”. | Not applicable |
| **Title** | The title is in the format ‘Topic / question: design/type of paper’ [not applicable to letters / editorials] | Ok – title page |
| **Abstract** | A **structured** abstract of no more than 400 words (+ optional tweetable abstract) appropriate to the design of the study is included **at the beginning** of your paper. [not applicable to letters / editorials] | The abstract is presented in the title page.  A copy is added into the manuscript. |
|  | No references are cited in the title / abstract. | OK |
| **Study registration** | Give any study registration number (e.g. ISRCTN), the registration date and date the first participant was recruited (if relevant) in both the **abstract and in the body** of the paper or state ‘not registered’*[[1]](#footnote-2). | The registration is presented into the main manuscript and available in the title page |
|  | For clinical trials (as defined by the ICMJE), registration occurred before the first participant was recruited. | OK |
| **Key words** | Give between four and ten key words that identify the paper's subject, purpose, method and focus. Use the Medical Subject Headings (MeSH®) or Cumulative Index to Nursing and Allied Health (CINAHL) terms (see <http://www.nlm.nih.gov/mesh/meshhome.html>). | P.2 of title page |
| **Contribution of the Paper statements** | Under the headings “What is already known” and “What this paper adds” give 2-3 (maximum) short, single sentence bullet points (each), summarising key contributions. No references are to be cited. [not applicable to letters / editorials] | P.2 of main manuscript |
| **Multiple publications** | Other published and in press accounts of the study from which data in this paper originate are referred to in the paper (author details can be redacted for review if desired) and the relationship between this and other publications from the same study is made clear in the paper. see below] | P.7 of the manuscript  And  P.17 of the manuscript.  References are redacted for review. |
|  | Full references to any such publications are provided for editors at the end of this checklist. | OK |
| **Ethical approval and informed consent** | Details of the ethical approval, including the body that granted it and reference number are included at **the end of you methods section** [**r**esearch papers only]. This should include confirmation of informed consent by participants and / or elaboration of the basis for any exception. | Ethics and funding are anonymized at the end of the manuscript  p.17 |
| **Statistical reporting** | Confidence intervals can be used as the basis for inference without reference to statistical significance & ‘p-values’. ***If*** reporting statistical significance tests: | Not applicable |
|  | - Exact p-values are stated to an appropriate degree of precision (typically no more than 3 decimal points). | Not applicable |
|  | - The corresponding measure of effect or association and confidence interval are reported with all significance tests (including in the abstract). | Not applicable |
|  | - The term ‘statistically significant’ (not just ‘significant’) is used to refer to the result of tests. | Not applicable |
|  | - p-values>0.05 are not used to conclude that there is no effect/association. | Not applicable |
| **Qualitative findings** | Where verbal data is used always include key quotations to support inferences and give meaningful (anonymous) individual subject identifiers for each quotation used. | Citations are added into the discussion |
| **Funding sources** | State sources of funding and the role of funders in the conduct of the research or include a statement ‘no external funding’ **at the end of the paper**. | Presented at the end of the manuscript  p.17 |
| **Conflict of interests** | State any actual or potential conflicts of interest in a section **at the end of the paper**. If there are none, include a statement “Conflicts of interest: none”. The substance of this declaration should match details provided in file(s) uploaded at submission. | Presented at the end of the manuscript  p.17 |
| **Please provide below references for any other publications based on data from the same study (including papers using data from the same participants reporting other outcomes or time points) and describe the relationship to the current study.**  *Please provide full references. To assist editors, upload copies of papers where the abstract / full text is not readily available (including those under review elsewhere, which will be treated in strict confidence). Where your paper is based on analysis of a publically available data set or is part of a series of publications from a large cohort study (or similar) you can be selective in the references you provide and give a more general account of how this paper relates to others but it is essential that editors are able to verify the unique contribution of the paper you are submitting.*  *If unsure about declarations we encourage you to err on the side of openness and suggest you consult Norman, I., Griffiths, P., 2008. Duplicate publication and 'salami slicing': Ethical issues and practical solutions. International Journal of Nursing Studies 45 (9), 1257-1260.*  **We do NOT require you to use this space to copy your paper’s reference list.** | | |
| Two publications relate to this methodological paper. First the Scoping review which was done at the early stage of the research project and followed by the study protocol. Both are available in Open Access.  Bobbink, P., Larkin, P. J., & Probst, S. (2020). Experiences of Venous Leg Ulcer persons following an individualised nurse-led education: Protocol for a qualitative study using a constructivist grounded theory approach. BMJ Open, 10(11), e042605. https://doi.org/10.1136/bmjopen-2020-042605  Bobbink, P., Pugliese, M.-T., Larkin, P., & Probst, S. (2020). Nurse-led patient education for persons suffering from a venous leg ulcer in outpatient’s clinics and homecare settings: A scoping review. Journal of Tissue Viability, 29(4), 297‑309. https://doi.org/10.1016/j.jtv.2020.08.006 | | |

*© Peter Griffiths, Ian Norman 2021*

*This work is licensed under a*[Creative Commons Attribution 4.0 International License.](http://creativecommons.org/licenses/by/4.0/)

1. While the journal endeavours to maintain a double blind-review process as far as possible, we give priority to transparent reporting and prospective registration. As it is important that reviewers are able to verify that reporting is complete and consistent with protocols to avoid (for example) selective outcome reporting or undocumented protocol changes, authors are not permitted to redact registration numbers for review. [↑](#footnote-ref-2)
