# Supplementary material for: Application and challenges of using a Constructivist Grounded Theory methodology to address an undertheorized clinical challenge: A discussion paper
Source: Int J Nurs Stud Adv. 2024 Apr 10;6:100199. doi: 10.1016/j.ijnsa.2024.100199 (PMC11080569; doi:10.1016/j.ijnsa.2024.100199)
Supplement: Supplementary file 3 [file mmc3.docx]

**CRediT authorship contribution statement**

Paul Bobbink : Writing – original draft, Conceptualization, Methodology

Philip Larkin : Writing – review & editing, Supervision, Methodology

Sebastian Probst : Writing – review & editing, Supervision, Methodology, Funding acquisition, Project administration
